# About being queer and Latinx in academia

**DOI:** 10.1016/j.eclinm.2022.101456

**Published:** 2022-05-20

**Authors:** Monica Malta

**Affiliations:** a Institute for Mental Health Policy Research, Centre for Addiction and Mental Health, Toronto, Ontario, Canada; b Faculty of Medicine, Department of Psychiatry, University of Toronto, Toronto, Ontario, Canada

I was born in Rio de Janeiro, Brazil. In my hometown, around 1.5 million people (25% of the population) live in slums. I grew up experiencing house instability and food insecurity - a reality faced by tens of millions of Brazilians. Never in my wildest dreams did I imagine that one day I would become a scientist and professor. Against all odds, I did.

Brazil is one of the most violent countries to be a women and the most violent country in the world for queer women. During the last five years, Brazil registered more than 15 murders of women every day.^1^ During the last 10 years, the country responded for over 40% of all murders of transgender and gender diverse persons in the world.^2^ In both tragic scenarios, the heavier burden is among People of Color.

My trajectory from poverty to academia was only possible because gender-based, affirmative policies were available. Because of those affirmative actions, I was able to enroll in and graduate from Ivy League universities. However, I was always an outlier. I was part of a minority surrounded by White peers and mostly White, male professors. On campus, security frequently stopped and demanded to see my badge, while my White peers could easily pass them - even without their own badges. At the university cafeteria, I was frequently stopped by students or faculty members complaining about an unclean bathroom or room. Those daily micro aggressions were common for Black and Latinx students - a clear demonstration that somehow we did not seem to belong.

Systemic racism on campus is still very common. Black, Latinx Ivy League students are still underrepresented as of today. Programs adopted to correct historic discrimination against the Black and Latinx community from Harvard, Yale and Princeton, among others, faced extreme opposition from the Trump Administration.^3^ Studies have shown that Black and Latinx borrow student loans at higher rates than their White peers. In the US, white households have at least 10 times the median income of Black and Latinx households, making White students more likely to receive help from their families to pay for their education. As a Latinx immigrant, first generation college student living in the US, without affirmative actions I would not be able to pay for my studies. Research has shown that the fallout of the student debt crisis is disproportionately borne by individual student loan borrowers of color, especially Black and Latinx borrowers.^4^ This disadvantaged scenario add to a preexisting racial/ethnic wage gap and harm upward mobility.

After my PhD graduation, I saw myself fighting (once again) systemic-level biases to continue in academia. According to the American Association of University Professors (AAUP),^5^ women, and particularly women of color, continue to face deep disparities in academia. Women faculty, especially women of color are underrepresented in academia's highest faculty positions – and overrepresented in its more precarious ones. The higher the academic rank, the lower the percentage of women - for Black and Latinx women the percent is even lower.

In the US, only 5.2% of full-time faculty members self-identify as Hispanic or Latinx and 6% identify as Black or African American, even though they're 17.5 and 12.7% of the country's population, respectively.^6^ The AAUP's analysis confirms that women faculty members, particularly Black and Latinx, continue to face unique challenges in academia with respect to employment, career advancement, salary, and job security, and that higher education is by no means immune from systemic racism.

Systemic racism and gender inequality in Academia is undeniable. Although some institutions have been more upfront to make necessary changes, there is still a long way to go. According to the Association of American Medical Colleges (AAMC), only 5.5% of medical school faculty are Hispanic, Latinx, or of Spanish origin; 3.6% are Black or African American; and 0.2% are Native American or Alaskan Native.

We do not need more trainings to address implicit bias. We need reliable, accessible and responsive structures supporting accountability. We need prompt response to denounces of micro aggressions and discrimination. Academia needs to assure diversity in search committees, support for students and faculties from racial and/or gender minorities to continue in academia, to cite just a few key strategies. The Ivy towers need to continue working to improve their commitments and strategies towards an environment where justice, equity, diversity and inclusion are not empty promises, but the reality.

## Contributors

Monica Malta.

## Declaration of interests

None.
